# *BdVRN1* Expression Confers Flowering Competency and Is Negatively Correlated with Freezing Tolerance in *Brachypodium distachyon*

**DOI:** 10.3389/fpls.2017.01107

**Published:** 2017-06-22

**Authors:** Ying Feng, Yanhai Yin, Shuizhang Fei

**Affiliations:** ^1^Interdepartmental Graduate Major in Genetics and Genomics, Iowa State University, AmesIA, United States; ^2^Department of Genetics, Development, and Cell Biology, Iowa State University, AmesIA, United States; ^3^Department of Horticulture, Iowa State University, AmesIA, United States

**Keywords:** vernalization, freezing tolerance, cold acclimation, flowering time, *Brachypodium*

## Abstract

Vernalization is an essential process by which many temperate plant species acquire competence for flowering. *Brachypodium distachyon* is a model plant for temperate grasses including many cool-season forage and turfgrasses and cereals. Grasses with spring growth habit do not require vernalization for flowering and are typically less winter hardy. Yet the connection between vernalization and freezing tolerance remain unclear. The diverse requirement of vernalization for flowering makes it an ideal choice for studying the relationship between vernalization and freezing tolerance. Here, we isolated and analyzed the spatial and temporal expression patterns of two vernalization related homologous genes, *BdVRN1* and *BdVRN3* in Bd21, a non-vernalization-requiring accession, and Bd29-1, an accession shown to be vernalization-requiring. We showed that expression of *BdVRN1* and *BdVRN3* is independent of vernalization in Bd21, but is vernalization dependent in Bd29-1. Moreover, vernalization-induced expression of *BdVRN1* appears to precede that of *BdVRN3* in Bd29-1. Bd21 RNAi knockdown mutants for *BdVRN1* conferred vernalization requirement for flowering, and reduced the expression of *BdVRN3.* Both Bd29-1 and the *BdVRN1* RNAi mutants of Bd21 exhibited reduced freezing tolerance upon vernalization treatment. Cold-responsive genes *BdCBF2*, *BdCBF3*, *BdCBF5*, *BdCBF6*, and *BdDREB2A* were all constitutively expressed at a high level in the *BdVRN1* RNAi mutants of Bd21. Taken together, our results suggest that expression of *BdVRN1* promotes flowering by upregulating *BdVRN3*, and gaining the competency for flowering reduces freezing tolerance in *Brachypodium*.

## Introduction

Flowering occurs as a result of a critical growth transition from vegetative to reproductive growth in plants. In plants with a winter growth habit, an exposure to a period of low temperature is essential to flowering induction, a process known as vernalization (Boss et al., 2004; Baurle and Dean, 2006). The long period of time required to fulfill the vernalization requirement assures that reproductive organs, which are more sensitive to chilling/freezing injuries than vegetative tissues, do not form in the middle of the winter (Kim et al., 2004; Balasubramanian et al., 2006; Baurle and Dean, 2006; Trevaskis et al., 2007).

The molecular mechanism of vernalization-induced flowering has been extensively studied in *Arabidopsis*, wheat, and barley (Amasino, 2005; Izawa, 2007; Schmitz and Amasino, 2007; Distelfeld et al., 2009; Srikanth and Schmid, 2011). *FLOWERING LOCUS T* (*FT*) in *Arabidopsis* is identified to be the “florigen” (Kardailsky et al., 1999; Kobayashi et al., 1999; Notaguchi et al., 2008), which encodes a small globular protein that is able to translocate from the leaves, through the phloem, to the shoot apex where it interacts with a bZIP transcription factor FLOWERING LOCUS D (FD) to activate the expression of the floral meristem identity gene *APETALA1* (*AP1*) for flowering (Abe et al., 2005; Wigge et al., 2005). In wheat and barley, *VERNALIZATION 3* (*VRN3*) and *VERNALIZATION 1* (*VRN1*) genes have been identified to be the homologs of the *Arabidopsis FT* and *AP1*, respectively (Danyluk et al., 2003; Murai et al., 2003; Trevaskis et al., 2003; Yan et al., 2003, 2006; Faure et al., 2007).

Previous studies, however, have shown that the vernalization pathways are not conserved between *Arabidopsis* and temperate cereals (Amasino, 2005; Trevaskis et al., 2007; Dennis and Peacock, 2009; Distelfeld et al., 2009; Greenup et al., 2009; Kim et al., 2009; Andres and Coupland, 2012). In *Arabidopsis*, *FLOWERING LOCUS C* (*FLC*), a MADS-box transcription factor gene, has been identified to be a negative regulator of flowering, which blocks the long-day induction of *FT* but itself is repressed by vernalization (Michaels and Amasino, 1999; Searle et al., 2006). In temperate cereals, no homologous gene of *FLC* has been found, but *VERNALIZATION 2* (*VRN2*) gene was identified as a flowering repressor in cereals, which encodes a zinc-finger and CCT (CONSTANS, CONSTANS-LIKE, TOC1) domain protein (ZCCT1) (Yan et al., 2004). *VRN2* is induced by long-day and can function like *FLC* to suppress long-day induction of *VRN3* (Yan et al., 2004, 2006). *VRN2* has also been reported to repress *VRN1* gene, but this repression is relieved by vernalization (Danyluk et al., 2003; Trevaskis et al., 2003; Yan et al., 2003). Vernalization induces the expression of *VRN1* which in turn repress *VRN2*, thereby releasing the repression of *VRN3* by *VRN2* (Yan et al., 2003, 2004, 2006). *VRN3* further elevates the expression of *VRN1* forming a signaling feedback loop of *VRN1-VRN2-VRN3-VRN1* that regulates flowering (Yan et al., 2004; Dubcovsky et al., 2006; Trevaskis et al., 2006). However, Shimada et al. (2009) proposed an alternative model of vernalization response in wheat. In a mutant that has null alleles of *VRN1*, the authors could not detect the expression of the *VRN3* gene and the mutant remained at the vegetative phase. In this alternative model, *VRN1* is directly upstream of *VRN3* instead of *VRN2* and up-regulates the *VRN3* expression with a vernalization treatment. *VRN3* in turn represses *VRN2*, releasing the inhibition of *VRN1* by *VRN2* (Shimada et al., 2009). The interrelationships among the *VRN* genes in temperate grasses remain to be investigated.

In general, plants with a vernalization-requiring winter growth habit have better freezing tolerance compared with plants having a spring growth habit that are non-vernalization-requiring, suggesting a link between vernalization requirement and freezing tolerance (Antikainen and Griffith, 1997; Limin and Fowler, 2006; Sandve et al., 2011; Chawade et al., 2012). However, whether and how vernalization is related to freezing tolerance remain to be investigated. Previous findings showed that vernalization pathway and cold acclimation pathway are interconnected (Liu et al., 2002; Seo et al., 2009; Dhillon et al., 2010; Lee et al., 2012). *SUPPERSSOR OF OVEREXPRESSION OF CONSTANS1* (*SOC1*), which encodes a MADS-box transcription factor, has been reported to regulate multiple floral induction pathways including vernalization, photoperiod, and autonomous (Onouchi et al., 2000; Samach et al., 2000). *SOC1* may also play an important role on regulation of cold acclimation, a period of exposure to low temperatures that results in a significant increase in freezing tolerance (acquired freezing tolerance). Cold acclimation induces the cold-responsive genes including the *C-repeat binding factor* (*CBFs*)/*Dehydrate Responsive Element Binding* (*DREB*) and *Cold Regulated* (*COR*) (Thomashow, 1999; Chinnusamy et al., 2007; Zhang et al., 2009, 2017; Chinnusamy et al., 2010). The *SOC1* knockout mutants increased the expression of cold-responsive genes such as *CBFs* and *COR* whereas *SOC1* overexpression mutants decreased the expression of these genes in *Arabidopsis* (Seo et al., 2009). In addition, heterologous expression of the wheat *VRN2* (*TaVRN2*) gene in *Arabidopsis* delayed flowering and enhanced freezing tolerance due to the accumulation of *CBF2*, *CBF3*, and *COR* genes (Diallo et al., 2010). In barley, genotype with the *vrn-1* allele had higher expression of *CBF* genes than genotypes with the *Vrn-1* allele, and *CBF* transcript abundance decreased after vernalization in the *vrn-1* genotype (Dhillon et al., 2010). In wheat, freezing tolerance and transcript abundance of several *CBF* and *COR* genes were much lower in a deletion mutant, *maintained vegetative phase* (mvp) in which *VRN1*, along with several other genes are deleted (Dhillon et al., 2010). Taken together, these results suggest that *VRN1* has a negative effect on the expression of *CBF* genes that influence freezing tolerance.

To better understand the mechanism of vernalization pathway and its relationship with freezing tolerance in temperate grasses, we used the monocot model plant, *Brachypodium distachyon* for our study. With its sequenced genome, a small physical stature, self-fertility, a short life cycle, an efficient transformation system and abundant natural variation in flowering habit, *Brachypodium* is well suited for studying the molecular mechanism of vernalization and its relationship with freezing tolerance (Draper et al., 2001; Vogel et al., 2010).

In this study, we isolated and analyzed the expression of three putative *VRN* genes in *Brachypodium* of either a non-vernalization-requiring (Bd21) or a vernalization-requiring accession (Bd29-1) and studied their freezing tolerance. We showed that *BdVRN1* and *BdVRN3* are induced by vernalization and are positive regulators of flowering in Bd29-1. The *BdVRN2*-like gene is likely not involved in the vernalization pathway in *Brachypodium*. Knockdown of *BdVRN1* in Bd21 also reduced the expression of *BdVRN3*, but had no effect on the expression of the *BdVRN2*-like gene and resulted in a dramatic non-flowering phenotype, which can be rescued by a vernalization treatment. Meanwhile, enhanced tolerance to freezing stress was observed in the RNAi mutants, accompanied by constitutive expression of several cold-responsive genes, *BdCBF2*, *BdCBF3*, *BdCBF5*, *BdCBF6*, and *DREB2A* at high levels. These results suggest that *BdVRN1* plays a critical role on flowering in vernalization pathway and its expression negatively affects the regulation of the cold-responsive genes and reduces freezing tolerance in *Brachypodium*.

## Materials and Methods

### Plant Materials and Growth Condition

Seeds of *B. distachyon* Bd21 (PI 254867) and Bd29-1(PI 639818) were sown in 6-inch pots containing Sun Gro Hort soil mix (Bellevue, WA, United States) in the greenhouse at 25°C, 16/8h (day/night) with an irradiance of 450 ± 50 μmol m^-2^ s^-1^.

### RNAi Vector Construction and Generation of RNAi Mutants

A fragment of *BdVRN1* (414 bp) gene was amplified by PCR (primers listed in Supplementary Table S2) with the addition of four bases of CACC at its 3′ end. PCR was performed as follows: 94°C for 5 min; 35 cycles of 94°C for 20 s, 59°C for 30 s, and 72°C for 50 s; final extension at 72°C for 1 min. The PCR product was separated on 1% agarose gel and extracted from the gel. Cleaned gene fragment was cloned into a Gateway^®^ entry vector pENTR^TM^/D-TOPO^®^ (Life Technologies, Grand Island, NY, United States) containing attL and attR recombination sites according to the manufacturer’s protocol. The Gateway^®^ compatible pANDA vector (Miki and Shimamoto, 2004) containing two cassettes in inverse orientation linked by a small fragment of the *GUS* gene was used as the destination vector for *BdVRN1*. The attL × attR reaction is mediated by Gateway^®^ LR Clonase^TM^ II enzyme mix (Life Technologies, Grand Island, NY, United States). Kanamycin (*nptII*) and hygromycin (*hpt*) resistance genes were used for the selection in bacteria and plants, respectively. The final binary vector pANDA::BdVRN1 was verified by Sanger sequencing (Supplementary Figure S1).

The pANDA::BdVRN1 vector was introduced into *Agrobacterium tumefaciens* C58C1 strain for transformation of *Brachypodium* following the protocol developed by Vogel and Hill (2008). The selection medium contained 150 mg L^-1^ Timentin (bioWORLD, Dublin, OH, United States) to suppress *Agrobacterium* growth and 40 mg L^-1^ hygromycin B (bioWORLD, Dublin, OH, United States) to kill untransformed calli. Two cycles of selection, each lasting 2 weeks were performed under dark at 23°C. Resistant calli were transferred into the regeneration medium containing Kinetin (KT) at 1 mg L^-1^ and hygromycin at 10 mg L^-1^ in a tissue culture chamber at 23°C, 16/8 h (light) with an irradiance of 120 μmol m^-2^ s^-1^. Shoots started to appear 7–10 days after the transfer. Rooting took place on a MS medium supplemented with 0.1 mg L^-1^ NAA and 10 mg L^-1^ hygromycin. Well-rooted plantlets were carefully moved into 6-inch pots containing Sun Gro Hort soil mix (Bellevue, WA, United States). Plants were grown in a growth chamber at 23°C, 16/8 h (day/night) with an irradiance of 400 ± 30 μmol m^-2^ s^-1^.

Transgenic plants were screened by PCR using primers (HPT-F 5′ GAATTCAGCGAGAGCCTG 3′, HPT-R 5′ ACATTGTTGGAGCCGAAA 3′) designed from the sequence of the hygromycin resistant gene present in the pANDA binary vector. Eight independent T_0_ lines were confirmed by PCR analyses. In two of the independent lines, RNAi-4 and RNAi-12, PCR-positive and negative plants segregated in a 3:1 ratio in the progenies, indicating the integration of the transgene occurred at a single locus in each of the two lines. Homozygous plants of T_2_ RNAi-4 and RNAi-12 lines were chosen for further analyses.

### Cold Acclimation, Vernalization Treatment, and Assessment of Freezing Tolerance

Cold acclimation was done by growing plants in growth chamber at a 4°C and 8/16 h (day/night) with an irradiance of 400 ± 30 μmol m^-2^ s^-1^ for 10 days. To determine the vernalization requirement for flowering, 4-week-old plants of Bd 21 or Bd29-1 accession were placed into a growth chamber at 4°C and 8/16 h (day/night) with an irradiance of 400 ± 30 μmol m^-2^ s^-1^ for 3, 6, 9, or 12 weeks before they were moved back to a greenhouse (25°C, 16/8 h day/night) with an irradiance of 450 ± 50 μmol m^-2^ s^-1^.

Freezing tolerance was evaluated for Bd21, Bd29-1and *BdVRN1* RNAi mutants. Five-week-old plants were treated with -5°C and 8/16 h (day/night) for 12 h. Following the freezing treatment, plants were moved into a growth chamber at 4°C for recovery for 1 day and were then transferred into a greenhouse (25°C, 16/8 h day/night) and evaluated for recovery 1 week later. Percentage of plants that survived the freezing test and regrew were recorded.

### Gene Expression Analysis by Semiquantitative and Real-Time RT-PCR Analysis

To examine the expression levels of *BdVRN* genes (Supplementary Table S1) in response to vernalization treatment, 4-week-old plants of each accession were placed into a growth chamber at 4°C and 8/16 h (day/night) with an irradiance of 400 ± 30 μmol m^-2^ s^-1^. Leaves and meristem were harvested separately 2, 4, 6, 8, 10, or 12 weeks following the conclusion of the vernalization treatment.

For semiquantitative RT-PCR analysis, total RNA was extracted from leaves or meristems with TRIzol^®^ Reagent (Invitrogen, Carlsbad, CA, United States). Reverse transcription was performed with the SuperScript^®^ III First-Strand Synthesis System for RT-PCR kit (Invitrogen, Carlsbad, CA, United States). PCR was performed with gene specific primers (See Supplementary Table S2 for primer information) with the following program: pre-denaturation at 94°C for 5 min; 25 cycles of denaturation at 94°C for 30 s, primer-annealing at 58°C for 30 s, elongation at 72°C for 50 s, and post elongation at 72°C for 5 min for all genes. The expression level of each gene was normalized to that of the *BdGAPDH* reference gene.

For Real-Time RT-PCR analysis, total RNA extraction and reverse transcription were done the same way as described for the semiquantitative RT-PCR analysis. Quantitative analyses were carried out on the Eco^®^ Real-Time PCR System (Illumina, Inc., San Diego, CA, United States) using the SYBR^®^ GreenER^TM^ qPCR SuperMix kit (Invitrogen, Carlsbad, CA, United States) according to the manufacturer’s instructions. The quantity of PCR products was determined at the end of each cycle by the Eco^®^ Software v4.0 (Illumina, Inc., San Diego, CA, United States). The expression level of each gene was normalized to that of the *BdGAPDH* gene, and the expression level for each gene in the wild type without treatment was set as 1.0. Primers used for PCR amplification are listed in Supplementary Table S3.

## Results

### *Brachypodium* Accessions Vary in Vernalization Requirement for Flowering

**FIGURE 1:**
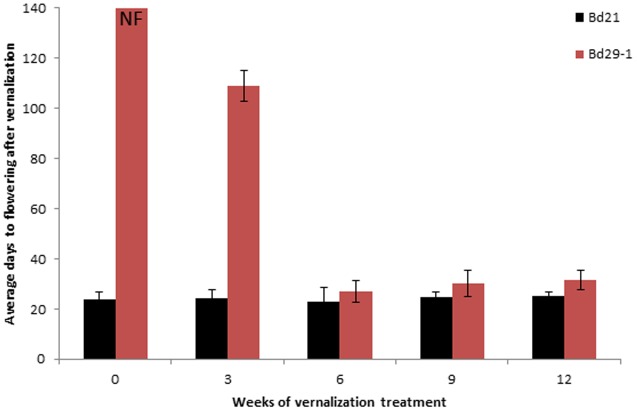
Average number of days required for flowering for the non-vernalization-requiring accession Bd21 and the vernalization-requiring accession Bd29-1 after vernalization treatment. Four-week-old seedlings (30 plants for each accession) were either not vernalized or vernalized for 3, 6, 9, or 12 weeks at 4°C and 8/16 h (day/night) in a growth chamber. After vernalization treatment, plants were transferred to a greenhouse (25°C, 16/8 h day/night). NF denotes no flowering observed. Average number of days to flowering indicate the number of days required for flowering following a vernalization treatment. Error bars represent the standard deviation from independent plants.

Time to flower was determined for accessions Bd29-1and Bd21 with or without vernalization treatment. Bd21 started flowering within 24 ± 3 days, regardless of the vernalization treatment (**Figure 1**), indicating vernalization treatment had no significant effect on accelerating flowering in Bd21. In contrast, without vernalization treatment Bd29-1 did not flower even after 140 days when record-taking was stopped. When subjected to a 3-week vernalization treatment, Bd29-1 started flowering after 109 ± 6.2 day. A 6-week vernalization treatment of Bd29-1 reduced the time to flower to just 27 ± 4.2 days. Longer than 6 weeks of vernalization treatment did not reduce the number of days to flowering further. Clearly, accession Bd29-1 requires vernalization treatment to flower.

### Vernalization Requirement of *Brachypodium* Is Strongly Associated with the Expression of *BdVRN* Genes

Three genes of *VRN1*, *VRN2*, and *VRN3* that regulate the vernalization requirement in wheat or barley have been characterized. Previous comparative genomic analysis showed that *Brachypodium* has a *VRN1* homolog, Bradi1g08340 (*BdVRN1*), located in a position co-linear to the rice (*OsMADS14*) and wheat (*TaVRN-1*) homologous genes, and a *VRN3* homolog, Bradi1g48830 (*BdVRN3*). Although there was no apparent homologous *VRN2*-like gene in *Brachypodium*, Bradi3g10010 is a member of the group IV CCT gene, an intermediate between the barley *HvCO9* and cereal *VRN2* genes in the phylogenetic tree (Higgins et al., 2010). For these reasons, we designated Bradi3g10010 as *BdVRN2*.

**FIGURE 2:**
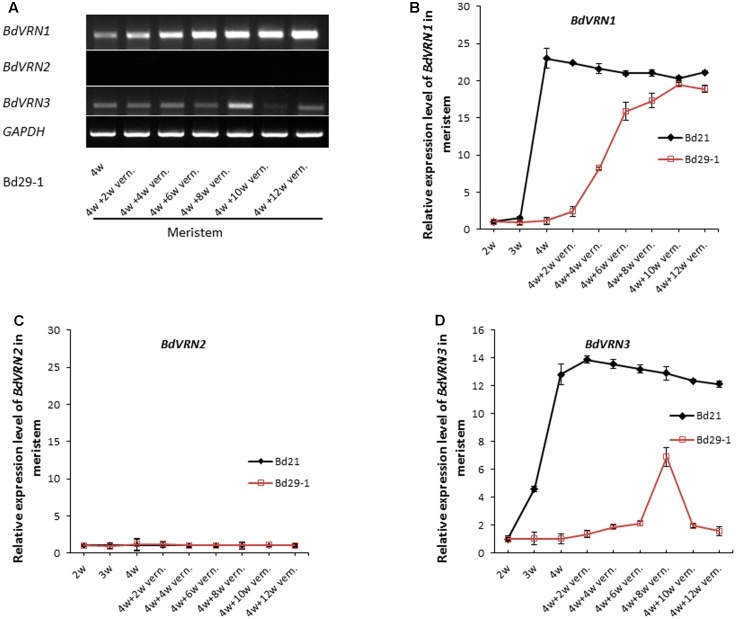
Expression of *BdVRN* genes in meristems of the non-vernalization-requiring Bd 21 and vernalization-requiring Bd29-1 accessions. **(A)** *BdVRN* gene expression in meristems of 4-week-old seedlings of Bd29-1 that were vernalized for 2, 4, 6, 8, 10, or 12 weeks. *BdGAPDH* was used as loading control. **(B–D)** Relative expression levels of *BdVRN1*, *BdVRN2*, and *BdVRN3* genes in meristems of Bd21 and Bd29-1. Prior to vernalization, plants were grown at a greenhouse (25°C, 16/8 h day/night). Four-week-old seedlings were transferred to a growth chamber at 4°C and 8/16 h (day/night) for vernalization treatment. Meristems were harvested after 2, 4, 6, 8, 10, or 12 weeks of vernalization treatment. Error bars represent the standard deviation from independent plants.

We next assayed the expression of the *BdVRN1*, *2* and *3* genes in both Bd21 and Bd29-1 with or without vernalization treatment. Without vernalization treatment, expression of *BdVRN1* expression remained at a low level in leaves and the meristem during the first 3 weeks following seed germination for both Bd21 and Bd29-1 (**Figure 2A** and Supplementary Figure S2). Four weeks after germination, *BdVRN1* expression was dramatically increased by 20-fold in the meristem in Bd21, whereas it remained unchanged in Bd29-1 (**Figures 2A,B**). Following various length of vernalization treatments, expression of *BdVRN1* in the meristem of Bd21 remained at a high level, whereas in Bd29-1 it was gradually increased to a level similar to that of Bd21 after 6 weeks of vernalization treatment (**Figures 2A,B**).

*BdVRN2* was expressed at a high level in leaves (Supplementary Figure S2A) but was expressed at a very low level in the meristem of both Bd21 and Bd29-1 (**Figure 2C**). Semiquantitative RT-PCR showed that *BdVRN2* was highly expressed in leaves of Bd21 with or without vernalization and its expression in Bd29 appeared to increase slightly after a 2-week vernalization; but longer vernalization did not increase further (Supplementary Figure S2B). Interestingly, the expression of *BdVRN2* in the meristem is not influenced by vernalization in both Bd21 and Bd29-1 (**Figure 2C** and Supplementary Figure S2B). In Bd21, expression of *BdVRN3* in the meristem increased continuously before vernalization treatment took place, and did not change significantly by vernalization treatment (**Figure 2D**). In contrast, in Bd29-1, the expression of *BdVRN3* in the meristem remained at a low level without vernalization, but increased gradually with vernalization treatment and increased by sixfolds when it reached the peak following a 8-week vernalization (**Figure 2D**).

### Knockdown of *BdVRN1* in Bd21 Conferred Vernalization Requirement

**FIGURE 3:**
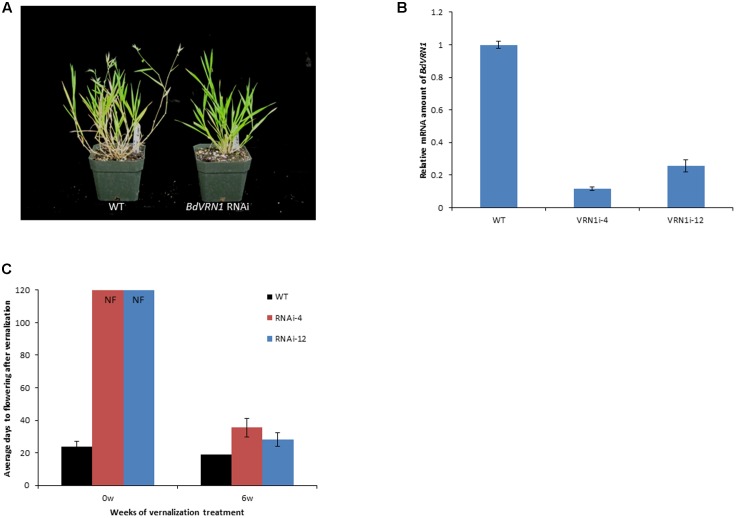
**(A)** Wild type (WT) (left) and *BdVRN1* RNAi mutant plant (right) 7 weeks after seed germination. **(B)** Real-Time RT-PCR analysis of the *BdVRN1* expression level in the WT, knockdown mutants of *BdVRN1* RNAi-4 and RNAi-12. **(C)** Average number of days required for flowering for the WT Bd21 and RNAi mutants with or without a 6-week vernalization treatment. Four-week-old seedlings (30 plants for each accession) were either not vernalized or vernalized for 6 weeks at 4°C and 8/16 h (day/night) in a growth chamber. After the vernalization treatment, plants were transferred to a greenhouse (25°C, 16/8 h day/night). NF denotes non-flowering phenotype. The average number of days to flowering indicates the number of days passed before flowering occurs following a vernalization treatment. Error bars represent the standard deviation from independent plants.

To determine whether *BdVRN1* indeed regulates flowering time, RNAi mutants of *BdVRN1* were created for Bd21. Eight independent T_0_ transgenic lines were confirmed by PCR analysis and two of them, RNAi-4 and RNAi-12 did not flower without vernalization (**Figures 3A,C**). Real-Time RT-PCR showed that the *BdVRN1* transcripts were reduced to 0.12 ± 0.02 in RNAi-4 and 0.26 ± 0.04 in RNAi-12 (**Figure 3B**). After a 6-week vernalization treatment, RNAi-4 and RNAi-12 mutants started flowering after 35.5 ± 5.6 days and 28.2 ± 8.3 days, respectively, compared to 19.2 ± 5.4 days for the wild type. The phenotypic change of Bd21 from non-vernalization-requiring to vernalization-requiring in the *BdVRN1* knockdown mutants strongly indicate that *BdVRN1* is of paramount importance to flowering induction in *Brachypodium*.

### Knockdown of *BdVRN1* in Bd21 Reduced the Expression of *BdVRN1* and *BdVRN3*, But Not *BdVRN2*

**FIGURE 4:**
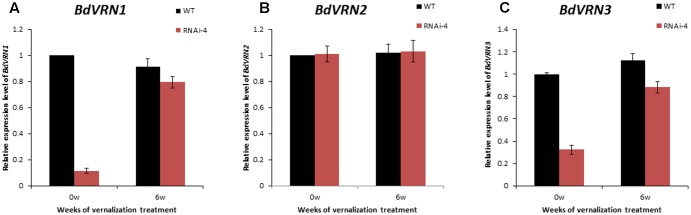
Expression analysis of *BdVRN1* **(A)**, *BdVRN2* **(B)**, and *BdVRN3* **(C)** genes in the WT (Bd21) and *BdVRN1* RNAi mutant. The relative expression levels of each gene were determined by Real-Time RT-PCR in leaf tissues. Prior to vernalization, plants were grown at a greenhouse (25°C, 16/8 h day/night). Four-week-old seedlings were then transferred to a growth chamber at 4°C (8/16 h day/night) for 0 or 6 weeks. *BdGAPDH* is a loading control. Error bars represent the standard deviation from independent plants.

To better understand how *BdVRN1* affects flowering at the molecular level, we analyzed the expression patterns of *BdVRN1*, *BdVRN2*, and *BdVRN3* in the RNAi-4 knockdown mutant. The expression of *BdVRN1* was significantly suppressed in 4-week-old seedlings of the RNAi mutant, but it increased significantly following a 6-week vernalization treatment, although it is still only half of the value observed for the wild type without cold acclimation (**Figure 4A**). Moreover, the expression level of *BdVRN3* also decreased in the *BdVRN1* mutants, but its expression was also elevated by vernalization treatment (**Figure 4C**). However, the expression of *BdVRN2* was not affected in the mutant (**Figure 4B**). These results indicated that upregulation of *BdVRN1* is positively correlated with *BdVRN3*, but not with *BdVRN2*.

### Decreased *BdVRN1* Expression Is Associated with Increased Freezing Tolerance Accompanied by Increased Expression of Cold-Responsive Genes

**FIGURE 5:**
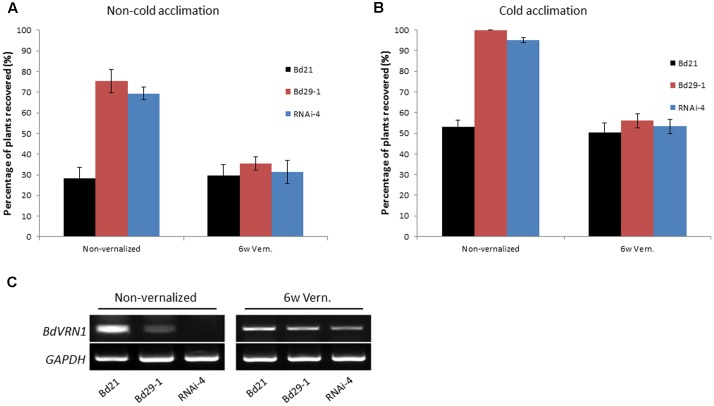
Freezing tolerance of vernalized or non-vernalized Bd21 and Bd29-1 with or without cold acclimation. **(A)** Freezing tolerance of non-vernalized or vernalized plants of Bd21, Bd29-1, and *BdVRN1* mutant RNAi-4 without cold acclimation. Freezing tolerance was assayed by subjecting plants to –5°C and 8/16 h (day/night) for 12 h in a growth chamber. Plants were then moved back into a greenhouse (25°C, 16/8 h day/night) and were examined for recovery a week later. The non-vernalized plants were 5-week-old. **(B)** Freezing tolerance of non-vernalized or vernalized plants of Bd21, Bd29-1, and RNAi-4 mutant following a 10 day cold acclimation. **(C)** Semiquantitative RT-PCR of *BdVRN1* gene in leaves from vernalized or non-vernalized plants of Bd21, Bd29-1, and RNAi-4 mutant. Leave tissues were collected before assessing freezing tolerance for each accession. Error bars represent the standard deviation from independent plants.

To determine the relationship between the vernalization requirement and freezing tolerance, we compared the freezing tolerance of Bd29-1 and Bd21. Without a vernalization treatment, Bd29-1 showed a much higher freezing tolerance than did Bd21 based on the percentage of plants that survived the freeze-thaw-regrowth test. When plants of both accessions were vernalized for 6 weeks, the recovery rate of Bd21 was nearly unchanged whereas that of Bd29-1 greatly decreased (**Figure 5A**). With a cold acclimation, both Bd21 and Bd29-1 had an increase in recovery rate compared with their non-cold acclimated counterparts (**Figure 5B**), which clearly indicated that both accessions have a well-defined cold acclimation mechanism to increase freezing tolerance upon an exposure to low temperatures. The result that vernalization treatment reduced the freezing tolerance of Bd29-1, but not Bd21suggested that vernalization requirement is negatively correlated with freezing tolerance. Furthermore the *BdVRN1* RNAi-4 mutant that gained vernalization requirement is also much more freezing tolerant than that of the wild type Bd21 with or without cold acclimation (**Figures 5A,B**). Semiquantitative RT-PCR experiments confirmed that without vernalization treatment Bd29-1 and the Bd21 RNAi-4 mutant had lower *BdVRN1* expression levels than the wild type Bd21, but a 6-week vernalization treatment increased the expression levels of *BdVRN1* in both Bd29-1 and the *BdVRN1* RNAi-4 mutant approaching to that in Bd21 (**Figure 5C**). This increase in *BdVRN1* expression coincides with the reduced freezing tolerance.

The regulation of gene expression in response to cold is quite complex with more than a 1000 genes are cold induced, but a critical group of them are organized into a cascade controlled through a regulatory hub involving cold-inducible *CBF* genes (Chinnusamy et al., 2007; Thomashow, 2010). The *CBF* genes encode members of the AP2/ERF family of transcription factors, which bind to the CRT/DRE regulatory elements in target genes of the CBF regulon and increase freezing tolerance (Jaglo-Ottosen et al., 1998; Liu et al., 1998; Catala et al., 2003; Dubouzet et al., 2003; Ito et al., 2006; Xiong and Fei, 2006; Lata and Prasad, 2011). For example, *CBF1* in *Arabidopsis* is a transcriptional activator for the *COR* genes, such as *COR47* and *COR414-TM1*, which are involved in cold and dehydration responses (Xiong et al., 2002; Sakuma et al., 2006; Griffith et al., 2007). DREB2A is a transcription factor induced by dehydration in *Arabidopsis*, rice, soybean, and maize (Simpson et al., 2003; Sakuma et al., 2006; Qin et al., 2007; Cui et al., 2011; Mizoi et al., 2013). The *RD* (*RESPONSIVE TO DESICCATION*) genes, for example *RD26* and *RD29B*, are induced by cold or drought stress through the regulation of the transcription factor DREBs (Yamaguchi-Shinozaki and Shinozaki, 1994; Nakashima et al., 2006; Msanne et al., 2011). To further characterize the relationship between *BdVRN1* gene and freezing tolerance, we analyzed the expression of some important cold-responsive genes by real-time RT-PCR in both the wild type Bd21 and the *BdVRN1* RNAi-4 mutants without vernalization treatment.

**FIGURE 6:**
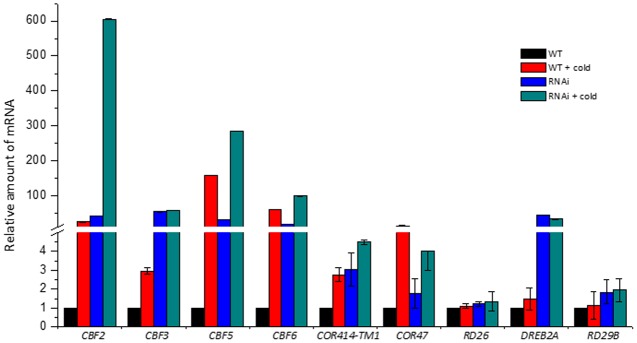
Real-Time RT-PCR analysis of the cold-responsive genes in the WT and RNAi mutant with or without cold treatment. Leaves were harvested 24 h after a 4°C cold treatment. The expression level of each gene was normalized to that of *BdGAPDH* gene, and the expression level for each gene in the WT without cold treatment was set to 1.0. Error bars represent the standard deviation from independent plants.

Previously, we isolated eight *CBF* genes (*CBF1* Bradi4g35630, *CBF2* Bradi4g35620, *CBF3* Bradi4g35650, *CBF4* Bradi4g35570, *CBF5* Bradi4g35580, *CBF6* Bradi4g35590, *CBF7* Bradi4g35600, and *CBF8* Bradi4g35650) from *Brachypodium* that are tandemly arranged in chromosome 4 and are all cold induced (not published). Here we compared their expression with four other important cold-responsive genes between the wild type Bd21 and the *BdVRN1* RNAi-4 mutants. We showed that *CBF2*, *CBF3*, *CBF5*, *CBF6*, *COR414-TM1*, and *COR47* were highly induced by 3- to 600-fold in both the wild type and the *BdVRN1* RNAi-4 mutants after a mere 24 h of cold treatment (**Figure 6**). More interestingly, *CBF*2, *CBF3*, *CBF5*, *CBF6*, and *DREB2A* were highly expressed in the *BdVRN1* RNAi-4 mutant without cold induction, and the constitutive expression of *CBF3* and *DREB2A* in the RNAi mutant was even higher than that of the cold-treated wild type. On the other hand, *RD26* and *RD29B* genes showed no noticeable changes in the RNAi mutant, compared to the wild type.

## Discussion

In this study, we examined the requirement for vernalization in *Brachypodium* accessions Bd29-1 and Bd21 and discovered that while Bd21 does not require vernalization for flowering, Bd29-1 requires vernalization. To understand the molecular basis underlining the differences in vernalization requirement between the two accessions, we isolated homologs of the three known cereal vernalization genes, *BdVRN1*, *BdVRN2*, and *BdVRN3* from both accessions, and analyzed their temporal expression patterns in leaves and the meristem. It was shown that the vernalization requirement is closely correlated with the expression patterns of the *BdVRN1* and *BdVRN3* genes, but not with the *BdVRN2* gene. Bd21 started flowering in approximately 7 weeks following seed germination, accompanied by accumulations of both *BdVRN1* and *BdVRN3* transcripts in the meristem regardless of vernalization treatment. In contrast, a minimum of 3-week vernalization is required for Bd29-1 to gain competence for flowering while 6 weeks or more significantly reduced the number of days for vernalized plants to flower. Accumulation of *BdVRN1* appears to precede the accumulation of *BdVRN3*. However, the expression of *BdVRN2* was not correlated to vernalization treatment in both Bd21 and Bd29-1. Therefore, we speculated that *BdVRN1* and *BdVRN3* positively regulate vernalization response of the vernalization-requiring accession Bd29-1. It is consistent with the results of a previous study that overexpression of FT1 (*VRN3*) caused extremely early flowering during shoot regeneration and downregulation of FT1 by RNA interference (RNAi) resulted in non-flowering *Brachypodium* (Lv et al., 2014). The loss-of-function mutants exhibited vernalization-requiring phenotype, similar to Bd29-1. Gene expression analysis showed that the expression of not only *BdVRN1* but also *BdVRN3* were suppressed in the mutants, however, expression of both were elevated by vernalization treatment. The expression of *BdVRN2* was not affected in the RNAi mutants with or without vernalization treatment, suggesting *BdVRN2* may not be a true homolog, or is not involved in flowering in *Brachypodium.* This is different from Ream et al. (2014) who reported that *BdVRN2* is induced during cold.

Our result is consistent with previous studies in wheat and barley that both *VRN1* and *VRN3* are induced by vernalization with a quantitative effect on the timing of flowering initiation (Danyluk et al., 2003; Murai et al., 2003; Yan et al., 2003, 2006). Different hypotheses have been proposed on the relationship between *VRN1* and *VRN3* in temperate cereals. Li and Dubcovsky (2008) posited *VRN3* regulate the *VRN1* expression in the vernalization pathway, while others suggested that *VRN1* is upstream of *VRN3* (Sasani et al., 2009; Shimada et al., 2009). Our results largely agree with the idea that *VRN1* is induced by vernalization and up-regulates the expression of *VRN3*.

Comparative genomic analysis suggested that *VRN2* gene has been lost in the Bd21 genome, similar to rice, which has no requirement for vernalization (Higgins et al., 2010). In our work, we chose a *CCT* gene grouped between *HvCO9* and *VRN2* in a phylogenetic tree as *BdVRN2*. We showed that *BdVRN2* was stably expressed in Bd21, which is consistent with the idea by comparative genomic analysis that this is a loss-of-function *BdVRN2* in Bd21. However, the *BdVRN2* was also stably expressed independent of the vernalization both in Bd29-1 and the *BdVRN1* RNAi mutants of Bd21, suggesting that *BdVRN2* is not involved in the vernalization pathway. This is different from wheat or barley, in which *VRN2* was characterized as a repressor of flowering and is regulated by both photoperiod and vernalization. Our result suggested that there are evolutionary difference between *Brachypodium* and temperate cereals. Interestingly a recent study identified a *FLC* homolog in *Brachypodium* that is a vernalization-regulated repressor (Sharma et al., 2016).

It is well observed that plants with vernalization requirement for flowering typically have better freezing tolerance than their non-vernalization-requiring close relatives (spring wheat vs. winter wheat or spring barley vs. winter barley), but the molecular link between them remains unclear. Previous works suggested that there might be a negative correlation between freezing tolerance and *VRN1* transcript in wheat (Liu et al., 2002; Danyluk et al., 2003; Limin and Fowler, 2006). The expression of *TaVRT*-1, a homolog of *AP1/VRN1* in wheat, was positively associated with vernalization treatment, and was negatively associated with the accumulation of *COR* genes and freezing tolerance (Danyluk et al., 2003). Expression studies showed that high levels of constitutive expression of some *CBF* genes in winter wheat cultivars confers a higher freezing tolerance compared to spring cultivars (Kobayashi et al., 2005; Badawi et al., 2007). In addition, the *VRN1* deletion mutant, *mvp*, increased freezing tolerance along with an increased expression level of several *CBF* and *COR* genes (Dhillon et al., 2010). A subsequent study found that the deletion of the *mvp* mutant include other genes besides *VRN1* such as *AGLG1*, *CYS*, and *PHYC* (Distelfeld and Dubcovsky, 2010). In the present study we showed that the vernalization-requiring Bd29-1 accession is more freezing tolerant than the non-vernalization-requiring Bd21, which corresponds to the different transcript levels of *BdVRN1* between the two accessions. Significantly, when we treated the Bd29-1 and Bd21with vernalization for 6 weeks, the freezing tolerance of Bd29-1 was greatly reduced, but that of Bd21 remained the same. In order to exclude other factors in regulating freezing tolerance, we compared the freezing tolerance between the non-vernalization-requiring Bd21 and its *VRN1* RNAi mutants which requires vernalization for flowering. Our results showed that freezing tolerance was enhanced in *BdVRN1* knockdown mutants relative to the wild type. Vernalization treatment of the RNAi mutants reduced their freezing tolerance. Moreover, several key cold-responsive genes, *BdCBF2*, *BdCBF3*, *BdCBF5*, *BdCBF6*, and *DREB2A*, are constitutively expressed at high levels in the knockdown mutants. Whether *BdVRN1* directly regulates the freezing tolerance pathway or function through interaction with other genes remains to be determined.

## Author Contributions

SF designed the experiment; YF carried out the experiment; YF, SF, and YY analyzed the data; and YF, SF, and YY wrote the manuscript.

## Conflict of Interest Statement

The authors declare that the research was conducted in the absence of any commercial or financial relationships that could be construed as a potential conflict of interest.
