# Supplementary material for: BdVRN1 Expression Confers Flowering Competency and Is Negatively Correlated with Freezing Tolerance in Brachypodium distachyon
Source: Front Plant Sci. 2017 Jun 22;8:1107. doi: 10.3389/fpls.2017.01107 (PMC5479923; doi:10.3389/fpls.2017.01107)
Supplement: Supplementary file 1 [file Data_Sheet_1.docx]

Table S1. Results of BLAST search of vernalization-related genes and cold stress inducible genes in *B. distachyon*

| Gene | Query gene accession no. | Species | Gene identified in *B. distachyon* |
| --- | --- | --- | --- |
| *VRN1* | AAZ76883 | *Triticum monococcum* | Bradi1g08340 |
| *VRN2* | AAS60241 | *Triticum monococcum* | Bradi3g10010 |
| *VRN3* | ABK32208 | *Triticum aestivum* | Bradi1g48830 |
| *CBF2* | ABI94367 | *Solanum tuberosum* | Bradi4g35620 |
| *CBF3* | ABE02655 | *Hordeum vulgare* | Bradi4g35650 |
| *CBF5* | ABV27084 | *Arabidopsis thaliana* | Bradi4g35600 |
| *CBF6* | AFM74029 | *Oryza sativa* | Bradi4g35590 |
| *COR414-TM1* | AEE31081 | *Arabidopsis thaliana* | Bradi2g16300 |
| *DREB2A* | Q0JQF7 | *Oryza sativa* | Bradi2g04000 |
| *RD29B* | BAA02375 | *Arabidopsis thaliana* | Bradi3g30320 |
| *RD26* | Q93VY3 | *Arabidopsis thaliana* | Bradi2g53260 |
| *COR47* | P31168 | *Arabidopsis thaliana* | Bradi5g10860 |

BLAST searches of *B. distachyon* genome were carried out on Phytozome V9.0 server (http://www.phytozome.com/search.php?show=text&method=Org_Bdistachyon) using the query amino acid sequences. Putative genes were isolated with primers designed from the coding region of each gene (Table S2).

Table S2. Sequences of primers used for semiquantitative RT-PCR

| Gene | Primer | Sequence (5’-3’) | Expected fragment size (bp) | |
| --- | --- | --- | --- | --- |
| *VRN1* | VRN1-F | ACAGAGGAGGCAGCAGGAC | 371 |  |
|  | VRN1-R | CCACGATTTATACAATGCTCGT |  | |
| *VRN2* | VRN2-F | ACGACTTCTTCCACCACAGC | 375 | |
|  | VRN2-R | AGCGGATCTGCTTCTCGTAG |  | |
| *VRN3* | VRN3-F | GACAGATATTCCAGGGACAACG | 300 | |
|  | VRN3-R | CGATGGGCCACGTATGTATT |  | |

Table S3. Sequences of primers used for Real-Time RT-PCR

| Gene | Primer name | Sequence (5’-3’) | Expected size (bp) | |
| --- | --- | --- | --- | --- |
| *VRN1* | VRN1-F | TGCTAGCAAGCTGACTACTTCG | 103 |  |
|  | VRN1-R | GGGTCACTGAAGGGACTGAA |  | |
| *VRN2* | VRN2-F | CACGATGCATGAGAGAGAGG | 101 | |
|  | VRN2-R | CGTAAGCTTTCCTGGACTCG |  | |
| *VRN3* | VRN3-F | GTCGCCGCCGTCTACTTC | 101 | |
|  | VRN3-R | CGATGGGCCACGTATGTATT |  | |
| *CBF2* | CBF2-F | GGAGTTCGAGTTCGACAAGG | 329 | |
|  | CBF2-R | GATCAGCCGAGACGAACAAT |  | |
| *CBF3* | 650-F | TTCCTCCCCTACATCCTCCT | 317 | |
|  | 650-R | CACTCGAACAGCTTCCCTTC |  | |
| *CBF5* | 600-F | CGAGCTGTGCACCTCAATAA | 323 | |
|  | 600-R | GAGCACCAAGGTGGAGGTAA |  | |
| *CBF6* | 590-F | GACATGATGAGCACCAGCAG | 331 | |
|  | 590-R | TGGAGGATCCTTCGATCTTG |  | |
| *COR414-TM1* | COR414-F | GGCATACCAGTCCAGCAGTT | 288 | |
|  | COR414-R | CCGCTCCATGTACTCACTCA |  | |
| *DREB2A* | DREB2A-F | ACCTCAGCACCTTCATTGCT | 254 | |
|  | DREB2A-R | TTGCCCTCCTGTTGGAATAC |  | |
| *RD29B* | RD29B-F | GGTGCTGAAGAAGGTCAAGG | 294 | |
|  | RD29B-R | CCCTTCTTCGTGTGATCCAT |  | |
| *RD26* | RD26-F | AAGTTCAACCCCTGGGATCT | 328 | |
|  | RD26-R | GCACCCACTCATCCAACTTT |  | |
| *COR47* | COR47-F | TCGAGAAGATCGATGGTGAA | 254 | |
|  | COR47-R | GTGGTAACCAGGCAGCTTGT |  | |
| *GAPDH* | GAPDH-F | GATCTGGTGTCCACCGACTT | 126 | |
|  | GAPDH-R | GCTGTAACCCCACTCGTTGT |  | |

Fig. S1 Schematic diagram of the VRN RNAi constructs. LB - left border, RB - right border, HPT - hygromycin phosphotransferase gene, HPT II – Kanamycin resistance gene, Ubq pro – Maize ubiquitin1 promoter, gus – gus linker sequence, nos - the nopaline synthase (nos) terminator.

Fig. S2 Expression analysis of *BdVRN* genes in leaves in Bd21 (non-vernalization-requiring) and Bd29-1 (vernalization-requiring). A. Semiquantitative PCR of *BdVRN* genes in leaves of 2, 3, or 4-week old seedlings and in meristems of 4-week old seedlings of Bd21. B. *BdVRN* genes expression in leaves of Bd29-1, 4-week old seedlings were vernalized for 2, 4, 6, 8, 10, and 12 weeks (*BdGAPDH* was used as loading control).
